# Case Report: Heparin-induced thrombocytopenia in a patient with COVID-19

**DOI:** 10.12688/f1000research.24974.2

**Published:** 2021-06-07

**Authors:** Ragia Aly, Sachin Gupta, Sorab Gupta, Balraj Singh, Abhinav Goyal, Sheila Kalathil

**Affiliations:** 1 Internal Medicine, Danbury Hospital, Danbury, CT, 06810, USA; 2 Internal Medicine, Tower Health Reading Hospital, Reading, PA, 19611, USA; 3 Internal Medicine, Albert Einstein Healthcare Network, Philadelphia, PA, 19141, USA; 4 Internal Medicine, St Joseph Regional Medical Center, Paterson, NJ, 07503, USA

**Keywords:** Heparin-induced thrombocytopenia, COVID- 19

## Abstract

With the spread of the novel coronavirus disease of 2019 (COVID-19) worldwide and associated high incidence of thromboembolic complications, the use of heparin is on the rise. It therefore is crucial to identify patients with contraindications for heparin. Heparin-induced thrombocytopenia (HIT) is a life-threatening complication of exposure to heparin. We report a 66-year-old woman, who was admitted to the hospital with COVID-19 infection. Her course was complicated by pulmonary embolism and dialysis catheter thrombosis. Our patient had a known history of HIT. Treatment of this patient with heparin would have been catastrophic. The COVID-19 pandemic has overwhelmed healthcare systems and is causing a global health crisis. Nevertheless, this case serves as a reminder of the importance of making every effort to obtain thorough history and review of records of every patient.

## Introduction

The severe acute respiratory illness caused by coronavirus disease of 2019 (COVID-19) disproportionately affects elderly and patients with multiple chronic comorbid conditions, making nursing home residents particularly vulnerable ^1^. Altered mental status is a common presentation of acute illness in this patient population, creating a challenge for obtaining an accurate medical history. In a Chinese study, 14% of patients with severe COVID-19 were noted to have impaired consciousness ^2^. There is also an alarmingly high incidence of thromboembolic complications, including pulmonary embolism, ischemic stroke and acute limb ischemia in COVID-19 patients ^3^. Here, we report a 66-year-old woman with a known history of heparin-induced thrombocytopenia (HIT), who was admitted to the hospital with COVID-19 infection.

## Case report

A 66-year-old woman with a medical history of end-stage renal disease on hemodialysis, type II diabetes mellitus, coronary artery disease and hypertension presented to the emergency department with fever, shortness of breath, cough, vomiting and abdominal pain. Vitals signs on presentation were as follows: temperature 101.2°F; heart rate of 102; blood pressure 152/76 mm hg; respiratory rate of 22/min; and oxygen saturation 93 on room air. On exam she was somnolent and confused and unable to provide previous medical history. Remaining physical exam was significant for mild tachypnea and tachycardia only. Nasopharyngeal swab was positive for COVID-19. The patient’s blood work was unremarkable except for mildly elevated troponin 0. 5 ng/ ml (0 and 0.4 ng/mL), d- dimer 800 ng/ ml (Normal is < 250 ng/mL), elevated blood urea nitrogen of 68 mg/dl (normal range 7–20 mg/dl) and creatinine of 11.1 mg/dl (normal range 0.8–1.2 mg/dl) and low platelet count of 119 ×10 ^3^/mcL (normal range, 150–400 × 10 ^3^/mcL). Of note, heparin was listed on the patient’s list of allergies, without further details.

**Figure 1.**
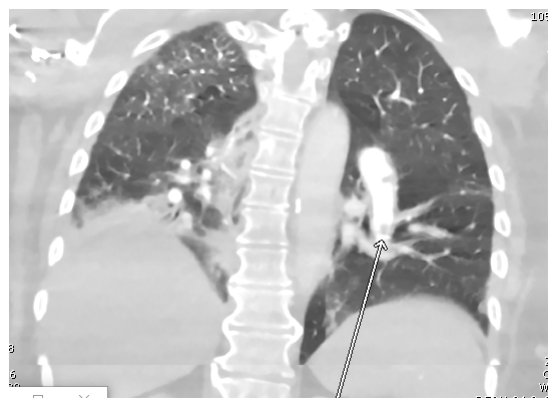
CTA chest showing an embolus in a branch of the left pulmonary artery (arrow).

The patient was admitted and started on hydroxychloroquine 400 mg every 12 hours for two doses followed by 200 mg every 12 hours for 5 days in addition to supportive measures, including acetaminophen 650 mg as needed for fever and supplemental oxygen. The patient’s regular hemodialysis schedule was also continued during her admission. A computed tomography pulmonary angiogram Figure 1 revealed pulmonary embolism in the left lower lobe, involving segmental and proximal subsegmental arteries without right heart strain or pulmonary infarction.

Given the history of allergy to heparin, the patient’s primary care provider was contacted to obtain further medical history and to confirm her heparin allergy. It was revealed that the patient was diagnosed with HIT a few weeks previously at a different facility for diabetic ketoacidosis. The patient was started on Argatroban infusion at starting rate of 2 mcg/kg/min, and this was adjusted regularly to maintain her activated partial thromboplastin time (aPTT) between 60 and 80 seconds.

The patient was hospitalized for a total of 14 days until she recovered from the acute illness, returned to her baseline mental status and was weaned off supplemental oxygen. her platelet count improved and she was transitioned to apixaban 2.5 mg twice daily (renally adjusted dose) to complete 3 months of anticoagulation. She returned to her skilled nursing facility on discharge, physical therapy was recommended on discharge due to generalized weakness and deconditioning. The patient had a virtual visit with the outpatient hematology clinic 6 weeks after discharge; she was tolerating treatment with apixaban with no adverse effects and her dyspnea had completely resolved.

## Discussion

COVID-19, caused by SARS-COV-2, was declared a worldwide epidemic by the World Health Organization on March 11th, 2020 ^4^. COVID-19 can present with a spectrum of clinical manifestations, including fever, myalgia, cough, dyspnea, and less frequently headache, diarrhea, nausea, and vomiting. Although respiratory symptoms predominate, thrombosis can also occur. The data collected from multiple centers worldwide have shown a high incidence of thromboembolic complications in patients with COVID-19. In a cohort study done by Helms *et al.,* 64 out of 150 patients, who developed acute respiratory distress syndrome (ARDS) secondary to SARS-COV-2 infection and were admitted to the Intensive Care Unit, developed thromboembolic complications, including pulmonary embolisms and ischemic strokes as well as circuit clotting during renal replacement therapy. The study also compared the incidence of pulmonary embolisms in patients with ARDS secondary to COVID-19 to that of patients with ARDS due to other etiologies, which was 11.7% compared to 2.1%, respectively ^3^. In light of the available data, patients admitted to hospital with moderate to severe COVID-19 pneumonia, especially those with abnormal coagulation parameters including elevated D-Dimer levels ^5^, are being treated with unfractionated heparin (UFH) or low molecular weight heparin (LMWH) in an effort to prevent and treat such potentially fatal complications ^6^. Patients with severe COVID-19 infection often present with acute encephalopathy. In an observational study done by Mao *et al.,* 36% of patients presenting with severe COVID-19 infections were encephalopathic on presentation ^7^. Other neurological complications were also reported, including, but not limited to, encephalitis, acute myelitis, and Guillain-Barre syndrome ^8^.

HIT is one of the most serious complications related to heparin use, with a mortality rate that can reach up to 20% ^9^. HIT is caused by autoantibody (IgG) that targets endogenous platelet factor 4 (PF4) and heparin complex. This PF4/IgG complex activates platelets and can trigger catastrophic venous and arterial thrombosis ^10^. The choice for treatment for acute HIT is non-heparin anticoagulants (argatroban, bivalirudin, danaparoid, fondaparinux along with direct oral anticoagulants).

As the incidence of COVID-19 associated thromboembolic complication increases in patients with severe illness, the use of UFH and LMWH has increased. Consequently, it becomes crucial to identify patients with contraindications to the use of heparin.

Patients with a history of HIT should avoid any forms of heparin exposure, which includes heparin flushes, heparin-bonded catheters, heparin-containing medications, such as prothrombin complex concentrates, some intravenous medication formulations, and some total parenteral nutrition preparations. With the increasing numbers of patients with severe COVID-19 pneumonia being admitted to intensive care units worldwide, the use of some of the previously mentioned heparin-containing treatments is on the rise. Patients with severe COVID-19 often present with encephalopathy and may require intubation and mechanical ventilation; both factors pose a challenge to obtaining complete medical history. Moreover, most hospitals have prohibited visitors during the pandemic, which is another barrier to obtaining history from patients’ families. A diagnosis of HIT is a serious diagnosis that must be documented accurately on patients’ medical records and be reported at every point of care transition.

## Conclusion

In conclusion, we report the case of a COVID-19 patient with a history of HIT, who developed pulmonary embolism, and the patient’s management. Our case and review of literature show that health care providers should be aware of life-threatening thromboembolic events associated with COVID-19, so that prompt and appropriate intervention can be undertaken. Our case also highlights the importance of history taking and thorough review of medical records in the COVID-19 pandemic to avoid catastrophic complications.

## Data availability

All data underlying the results are available as part of the article and no additional source data are required.

## Consent

Written informed consent for the publication of the case report and any associated images was obtained from the patient.
