# Supplementary material for: Case Report: Heparin-induced thrombocytopenia in a patient with COVID-19
Source: F1000Res. 2021 Jun 7;9:704. Originally published 2020 Jul 14. [Version 2] doi: 10.12688/f1000research.24974.2 (PMC8218025; doi:10.12688/f1000research.24974.2)
Supplement: Peer review history

# Peer review: Reviewer response for version 2

**DOI:** 10.5256/f1000research.57364.r86900

**Published:** 2021-06-21

**Authors:** Pooja Gogia

**Affiliations:** 1 Department of Medicine, Saint Peter's University Hospita, New Brunswick, NJ, USA; 2 Rutgers-Robertwood Johnson Hospital, New Brunswick, NJ, USA

The changes have been incorporated.

Are enough details provided of any physical examination and diagnostic tests, treatment given and outcomes?

Partly

Is the case presented with sufficient detail to be useful for other practitioners?

Partly

Is sufficient discussion included of the importance of the findings and their relevance to future understanding of disease processes, diagnosis or treatment?

Partly

Is the background of the case’s history and progression described in sufficient detail?

Partly

Reviewer Expertise:

Oncology, Hematology

I confirm that I have read this submission and believe that I have an appropriate level of expertise to confirm that it is of an acceptable scientific standard.

# Peer review: Reviewer response for version 1

**DOI:** 10.5256/f1000research.27551.r77377

**Published:** 2021-02-15

**Authors:** Pooja Gogia

**Affiliations:** 1 Department of Medicine, Saint Peter's University Hospita, New Brunswick, NJ, USA; 2 Rutgers-Robertwood Johnson Hospital, New Brunswick, NJ, USA

The authors should include more detailed case report presentation including physical examination, pertinent labs including d-dimer, troponin and inflammatory markers. Chest Xray should be included as well. 

 The discussion should also be more comprehensive and be focusing on treatment options in patients with Heparin induced thrombocytopenia. A brief discussion about her prior HIT diagnosis and the reason for her prior admission would be appropriate.

Are enough details provided of any physical examination and diagnostic tests, treatment given and outcomes?

Partly

Is the case presented with sufficient detail to be useful for other practitioners?

Partly

Is sufficient discussion included of the importance of the findings and their relevance to future understanding of disease processes, diagnosis or treatment?

Partly

Is the background of the case’s history and progression described in sufficient detail?

Partly

Reviewer Expertise:

Oncology, Hematology

I confirm that I have read this submission and believe that I have an appropriate level of expertise to confirm that it is of an acceptable scientific standard, however I have significant reservations, as outlined above.

# Response

**Published:** 2021-05-29

**Authors:** Sachin Gupta

**Affiliations:** docgupta1@gmail.com, USA

Author comments

 Thanks a lot for reviewing my manuscript. I made the changes as recommended. I have included  detailed case report presentation including physical examination, pertinent labs including inflammatory markers and CT PE findings. HIT management along with reason of her prior admission were also discussed in detail.

# Peer review: Reviewer response for version 1

**DOI:** 10.5256/f1000research.27551.r67133

**Published:** 2020-09-03

**Authors:** Vikramaditya Dumpa

**Affiliations:** 1 Pediatrics, New York University (NYU) Winthrop Hospital, Mineola, NY, USA

The authors describe a case of an elderly woman with multiple co-morbidities affected by COVID-19. The patient had thromboembolic complications resulting from COVID-19, and with a known history of heparin-induced thrombocytopenia, received treatment with other drugs thus avoiding further complications, This is a very timely and useful case report reminding health care providers to be conscious of potential allergies to drugs being used in the management of COVID-19 patients.

Are enough details provided of any physical examination and diagnostic tests, treatment given and outcomes?

Yes

Is the case presented with sufficient detail to be useful for other practitioners?

Yes

Is sufficient discussion included of the importance of the findings and their relevance to future understanding of disease processes, diagnosis or treatment?

Yes

Is the background of the case’s history and progression described in sufficient detail?

Yes

Reviewer Expertise:

Hematology, Pediatrics

I confirm that I have read this submission and believe that I have an appropriate level of expertise to confirm that it is of an acceptable scientific standard.
